# Alignment Layer of Liquid Crystal Using Plant-Based Isoeugenol-Substituted Polystyrene

**DOI:** 10.3390/polym13040547

**Published:** 2021-02-12

**Authors:** DaEun Yang, Kyutae Seo, Hyo Kang

**Affiliations:** BK-21 Four Graduate Program, Department of Chemical Engineering, Dong-A University, 37 Nakdong Daero 550beon-gil, Saha-gu, Busan 604-714, Korea; 1830133@donga.ac.kr (D.Y.); kyutae@donga.ac.kr (K.S.)

**Keywords:** liquid crystal, alignment, vertical, polystyrene, isoeugenol

## Abstract

We synthesized a series of renewable and plant-based isoeugenol-substituted polystyrenes (PIEU#, # = 100, 80, 60, 40, and 20, where # is the molar percent content of isoeugenol moiety), using polymer modification reactions to study their liquid crystal (LC) alignment behavior. In general, the LC cells fabricated using polymer film with a higher molar content of isoeugenol side groups showed vertical LC alignment behavior. This alignment behavior was well related to the surface energy value of the polymer layer. For example, vertical alignments were observed when the polar surface energy value of the polymer was smaller than approximately 3.59 mJ/m^2^, generated by the nonpolar isoeugenol moiety with long and bulky carbon groups. Good alignment stability at 100 °C and under ultraviolet (UV) irradiation of 15 J/cm^2^ was observed for the LC cells fabricated using PIEU100 as a LC alignment layer. Therefore, renewable isoeugenol-based materials can be used to produce an eco-friendly vertical LC alignment system.

## 1. Introduction

Approximately 380 million tons of plastic are produced globally each year, and up to 13 million tons are released into water streams annually [1,2]. By 2050, 330 million tons of plastic are predicted to be released in rivers and oceans. Since only about 9 percent of plastic is recycled, much of the remainder pollutes the environment or sits in landfills [2,3,4]. Plastic can take up to 500 years to decompose while leaching toxic chemicals into the ground [5,6,7]. It is usually discarded after use and has a negative impact on the environment because of its extremely low biodegradability [8]. There is increasing scientific and societal concern about the effects of microplastics (MPs), commonly defined as plastic particles with a size below 5 mm [9,10,11]. The negative influence of MPs on the environment has been widely reported, and the toxicity of MPs in water has been demonstrated [12,13,14,15]. Bioplastics, which are bio-based, biodegradable, or both, have the same properties as conventional plastics and offer additional advantages [16,17,18]. These include reduced use of fossil fuel resources and lower carbon dioxide emissions [19,20]. Bioplastics are also less toxic and do not contain health-damaging additives, such as phthalates or bisphenol-A [21,22,23]. Bioplastics are considered a promising solution to concerns related to microplastics because they are environmentally friendly [24,25,26,27]. In this study, polymers containing bio-resources such as isoeugenol were prepared as a way to address pollution problems [28].

Isoeugenol belongs to the group of phenylpropenes, is found in essential oils of plants such as ylang-ylang (*Cananga odorata*), and is a component of wood smoke [29,30]. As one of the most important components of natural flavors, isoeugenol (2-methoxy-4-(prop-1-en-1-yl)phenol) has been widely used in perfumes, soaps, detergents, air fresheners, and as a flavoring agent in cosmetics and food additives [31,32,33,34,35]. Because isoeugenol-constituted phenolic groups are known to have antioxidant and antibacterial functions, they can provide oxidation protection and bacterial fouling resistance [36,37,38,39,40]. For example, free radicals and active oxygen species have been associated with cardiovascular and inflammatory diseases, and even play a role in cancer and aging [41,42,43]. The antioxidant activity of isoeugenol can be attributed to its hydrogen atom and the electron-donating ability of ABTS (2,2′-azino-bis(3-ethylbenzothiazoline-6-sulfonic acid)) with radical-scavenging activity in isoeugenol [44,45,46,47]. The reducing power of isoeugenol has been noted to possess antioxidative potential because it breaks free radical chains and donates a hydrogen atom [48,49]. Moreover, isoeugenol can provide highly effective antimicrobial coatings that prevent bacterial attachment and apply bactericidal surfaces [50,51]. Isoeugenol can be used to add hydroxy groups to the surfaces of substrates, such as polymers and metals for a wide range of surface coating applications [52]. In addition, isoeugenol can be modified into polymers and used in the food industry and new pharmaceutical developments, and the antifungal activity of isoeugenol-binding materials can be manifested for pharmacological applications by docking isoeugenol to organic molecules [53,54,55].

Liquid crystal (LC) molecules have been extensively studied owing to their unique and attractive characteristics, such as solid-like ordering and liquid-like fluidity [56]. LC molecules have been known to exhibit anisotropic physico-chemical characteristics, such as optical and dielectric anisotropy, generated by external stimuli because of their interesting chemical structures [57]. Therefore, LC molecules have been used in various areas, such as information technology, energy and environmental technology, nanotechnology, and biotechnology, using their unique physico-chemical properties [58,59]. For example, LC molecules such as transmissive LCs, nematic LCs, reflective LCs, and cholesteric LCs have been widely used in the display industry [60]. Technologies to align LC molecules in the same direction on a substrate can be used in a variety of fields such as chemistry [61], physics [62], biology [63], and nanotechnology [64], by inducing interactions between LC molecules and surfaces on the substrate. Mechanical rubbing is a common method used to produce uniform orientation of LC molecules [65]. Polyimide (PI) derivatives are most commonly used as LC aligning materials using the rubbing technique because they provide stable LC-aligning properties [66]. Polyimide derivatives having alkyl and/or alkyloxy side groups, such as polyimide derivatives containing octadecyl and *n*-decyloxybiphenyloxy side chains, exhibit vertical LC aligning behaviors [67,68,69]. Although polyimide derivatives have been widely employed in the display industry, polyimide precursors are treated at high temperatures in order to produce polyimide film, and the film itself shows a yellowish color. Therefore, long-alkyl-chain-modified polystyrene (PS) derivatives have been synthesized as alternatives to polyimide derivatives to produce vertical LC orientation layers. The benefits of using PS instead of PI derivatives include high optical transparency, and more convenient processing and fabrication. In addition, PS derivatives with enhanced thermal properties can be obtained through copolymerization with methyl methacrylate [70], acrylonitrile [71], divinylbenzene [72], and trivinylbenzene [73], etc., or by incorporating specific moieties or functional groups into the side chain of polystyrene. For example, the introduction of coumarin as a dye [74], oryzanol as a plant extraction resource [75], and the imide moieties [76] into the side chain of polystyrene improves the *T*_g_ value. PS derivatives have been enhanced via a simple polymer reaction to produce vertical LC orientation layers for next-generation applications owing to their advantages, such as low-temperature processability and good solubility in many organic solvents [77]. The molecular orientation of polymers, such as isomeric groups on polymeric surfaces, is an important factor in inducing LC aligning behavior because of the interactions and/or steric repulsions between the polymer surface and the LC molecule [78,79]. In recent studies, much research has been conducted on liquid crystal alignment methods in order to overcome the limitations of mechanical rubbing using polyimide. For example, among the non-contact methods, photoalignment and photopatterning technology, which are promising alignment methods, have been noted as alternatives to rubbing technology. Another example is self-alignment technology or PI layer-less orientation technology, which introduces dopants such as nanoparticles and surfactants into the LC medium for next-generation applications.

In this study, bio-based isoeugenol-substituted polystyrenes (PIEU#) were synthesized to obtain vertical alignment of LCs and to study the effect of the molar content of the isoeugenyl side groups on LC alignment behavior. We selected isoeugenol, with its phenylpropene structure, as a modifier into the side chain of the polystyrene in order to investigate LC alignment behavior. Plant-based isoeugenol is not only effective and inexpensive for fabrication, but also safe and environmentally friendly. The optical and electrical properties of LC cells fabricated using renewable polymer films were also investigated.

## 2. Materials and Methods

### 2.1. Materials

4-Chloromethylstyrene, isoeugenol, and potassium carbonate were purchased from Aldrich Chemical Co., and a nematic LC, MLC-6608 (*n*_e_ = 1.5586, *n*_o_ = 1.4756, and Δ*ε* = −4.2, where *n*_e_, *n*_o_, and Δ*ε* represent the extraordinary refractive index, ordinary refractive index, and dielectric anisotropy, respectively) was purchased from Merck Co (Pyeongtaek, Korea). *N*,*N*′-Dimethylacetamide (DMAc) and ethanol were dried over molecular sieves (4 Å). Tetrahydrofuran (THF) was dried by refluxing with benzophenone and sodium, followed by distillation. 4-Chloromethylstyrene was purified by column chromatography on silica gel using hexane as an eluent to remove any impurities and inhibitors (tert-butylcatechol and nitroparaffin). Poly(4-chloromethylstyrene) (PCMS) was synthesized through conventional free radical polymerization of 4-chloromethylstyrene using 2,2′-azoisobutyronitrile (AIBN) under a nitrogen atmosphere. AIBN (Junsei Chemical Co., Ltd., Tokyo, Japan) was used as the initiator. AIBN was purified by crystallization using methanol. All other reagents and solvents were used as received.

### 2.2. Preparation of Isoeugenol-Modified Polystyrene

The following procedure was used to synthesize all of the isoeugenol-substituted polystyrenes, PIEU#, where # represents the molar content (in %) of isoeugenol moiety in the side group. The synthesis of isoeugenol-substituted polystyrene (PIEU100) is provided as an example. A mixture of isoeugenol (0.49 g, 2.95 mmol, 150 mol% compared with PCMS) and potassium carbonate (0.50 g, 3.54 mmol) in *N*,*N*′-dimethylacetamide (DMAc, 30 mL) was heated to 75 °C. A PCMS solution (0.3 g, 1.97 mmol) in DMAc (20 mL) was added to the mixture, which was then magnetically stirred at 70 °C for 24 h under a nitrogen atmosphere. The mixture was cooled to room temperature and poured into methanol to obtain a white precipitate. The precipitate was further purified by several reprecipitations from the DMAc solution into methanol and then washed with hot methanol to remove potassium carbonate and any remaining salts. PIEU100 was obtained in yields above 80% after drying overnight in a vacuum. The degree of substitution of the chloromethyl group with the isoeugenyl methyl ether group was found to be almost 100% within experimental error.

PIEU100 ^1^H NMR (CDCl_3_): *δ* = 0.91–1.42 (m, 3H, –*CH*_2_–*CH*–Ph–), 1.61–1.83 (d, 3H, –CH=CH–*CH_3_*), 3.51–3.92 (s, 3H, –CH_2_–O–Ph(*OCH_3_*)–CH=), 4.61–5.11 (s, 2H, –Ph–*CH*_2_–O–), 5.94–6.13 (m, 1H, –CH=*CH*–CH_3_), 6.12–6.31 (d, 1H, –Ph–*CH*=CH–), 6.31–7.23 (m, 7H, –CH_2_–CH–*PhH*–CH_2_–O–*PhH*(OCH_3_)–CH_2_=).

Other polystyrene derivatives containing isoeugenol side groups were synthesized using the same procedure used for the preparation of PIEU100, except for differing amounts of isoeugenol in the reaction. For example, PIEU80, PIEU60, PIEU40, and PIEU20 were prepared with 0.27 g (1.92 mmol), 0.19 g (1.15 mmol), 0.14 g (0.77 mmol), and 0.06 g (0.39 mmol) of isoeugenol, respectively, using slight excess amounts of potassium carbonate (0.50 g, 3.54 mmol, 180 mol% compared with PCMS).

PIEU 80 ^1^H NMR (CDCl_3_): *δ* = 0.93–1.44 (m, 3H, –CH_2_–CH–Ph–), 1.62–1.82 (d, 3H, –CH=CH–*CH_3_*), 3.41–3.82 (s, 3H, –CH_2_–O–Ph(*OCH_3_*)–CH=), 4.38–4.61 (s, 2H, –Ph–*CH*_2_–Cl), 4.61–5.13 (s, 2H, –Ph–*CH*_2_–O–), 5.91–6.12 (m, 1H, –CH=*CH*–CH_3_), 6.09–6.31 (d, 1H, –Ph–*CH*=CH–), 6.32–7.24 (m, 4H, –CH_2_–CH–*PhH*–CH_2_–Cl), 6.32–7.24 (m, 7H, –CH_2_–CH–*PhH*–CH_2_–O–*PhH*(OCH_3_)–CH_2_=).

PIEU 60 ^1^H NMR (CDCl_3_): *δ* = 0.93–1.43 (m, 3H, –CH_2_–CH–Ph–), 1.61–1.83 (d, 3H, –CH=CH–*CH_3_*), 3.41–3.83 (s, 3H, –CH_2_–O–Ph(*OCH_3_*)–CH=), 4.38–4.61 (s, 2H, –Ph–*CH*_2_–Cl), 4.61–5.14 (s, 2H, –Ph–*CH*_2_–O–), 5.92–6.13 (m, 1H, –CH=*CH*–CH_3_), 6.08–6.32 (m, 1H, –Ph–*CH*=CH–), 6.31–7.25 (m, 4H, –CH_2_–CH–*PhH*–CH_2_–Cl), 6.31–7.25 (m, 7H, CH_2_–CH–*PhH*–CH_2_–O–*PhH*(OCH_3_)–CH_2_=).

PIEU 40 ^1^H NMR (CDCl_3_): *δ* = 0.93–1.43 (m, 3H, –CH_2_–CH–Ph–), 1.62–1.82 (d, 3H, –CH=CH–*CH_3_*), 3.41–3.83 (s, 3H, –CH_2_–O–Ph(*OCH_3_*)–CH=), 4.38–4.62 (s, 2H, –Ph–*CH*_2_–Cl), 4.62–5.11 (s, 2H, –Ph–*CH*_2_–O–), 5.93–6.14 (m, 1H, –CH=*CH*–CH_3_), 6.09–6.33 (m, 1H, –Ph–*CH*=CH–), 6.34–7.25 (m, 4H, –CH_2_–CH–*PhH*–CH_2_–Cl), 6.34–7.25 (m, 7H, –CH_2_–CH–*PhH*–CH_2_–O–*PhH*(OCH_3_)–CH_2_=).

PIEU 20 ^1^H NMR (CDCl_3_): *δ* = 0.95–1.45 (m, 3H, –CH_2_–CH–Ph–), 1.63–1.82 (d, 3H, –CH=CH–*CH_3_*), 3.42–3.83 (s, 3H, –CH_2_–O–Ph(*OCH_3_*)–CH=), 4.38–4.62 (s, 2H, –Ph–*CH*_2_–Cl), 4.62–5.14 (s, 2H, –Ph–*CH*_2_–O–), 5.93–6.11 (m, 1H, –CH=*CH*–CH_3_), 6.07–6.36 (m, 1H, –Ph–*CH*=CH–), 6.34–7.24 (m, 4H, –CH_2_–CH–*PhH*–CH_2_–Cl), 6.34–7.24 (m, 7H, –CH_2_–CH–*PhH*–CH_2_–O–*PhH*(OCH_3_)–CH_2_=).

### 2.3. Film Preparation and LC Cell Assembly

Solutions of PIEU# in THF (1 wt.%) were prepared. These solutions were filtered using a poly(tetrafluoroethylene) (PTFE) membrane with a pore size of 0.45 μm. Thin films of the polymers were prepared by spin-coating (2000 rpm, 90 s) onto glass substrates. The LC cells were fabricated using a polymer film on glass slides. The LC cells were constructed by assembling films together using spacers with a thickness of 4.25 μm. The cells were filled with a nematic LC, MLC-6608. The manufactured LC cells were sealed using epoxy glue.

### 2.4. Instrumentation

Proton nuclear magnetic resonance (^1^H NMR) measurements were carried out on a Bruker AVANCE spectrometer at 300 MHz. Differential scanning calorimetry (DSC) measurements were carried out on a TA Instruments Q-10 at heating and cooling rates of 10 °C∙min^−1^ under a nitrogen atmosphere. The contact angles of distilled water, methylene iodide, formamide, and ethylene glycol on the polymer films were determined with a Kruss DSA10 contact angle analyzer equipped with drop shape analysis software. Surface energy values were calculated using the Owens–Wendt equation:

(1) $$\gamma_{sl}=\gamma_{s}+\gamma_{l}-2{\left(\gamma_{s}{}^{d}\gamma_{l}{}^{d}\right)}^{1/2}-2{\left(\gamma_{s}{}^{p}\gamma_{l}{}^{p}\right)}^{1/2}$$

where *γ_l_* is the surface energy of the liquid, *γ_sl_* is the interfacial energy of the solid/liquid interface, and *γ_s_* is the surface energy of the solid. *γ_s_^d^* and *γ_s_^p^* are the dispersive and polar components of the surface energy of the solid, respectively. *γ_l_^d^* and *γ_l_^p^* are the dispersive and polar components of the surface tension of the liquid, respectively. *γ_l_^d^* and *γ_l_^p^* are known for the test liquids, and *γ_s_^d^* and *γ_s_^p^* can be calculated from the measured static contact angles [80]. Polarized optical microscopy (POM) images of the LC cell were taken using an optical microscope (Nikon, ECLIPSE E600 POL, Tokyo, Japan) equipped with a polarizer and digital camera (Nikon, COOLPIX995, Tokyo, Japan).

## 3. Results

### 3.1. Synthesis and Characterization of Isoeugenol-Modified Polystyrene

**Figure 1:**
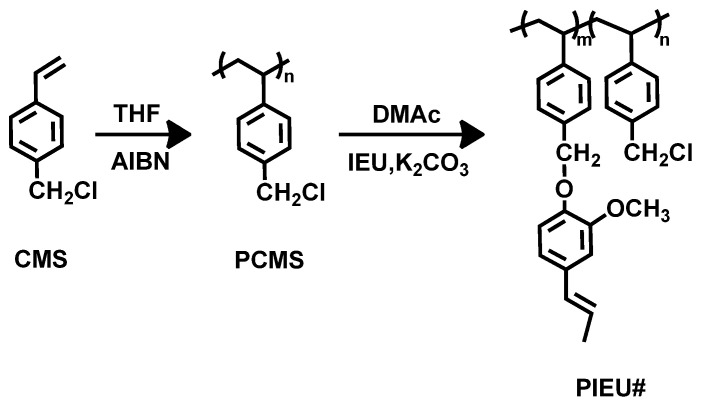
Synthetic route to isoeugenol modified polystyrene (PIEU#), where # indicates the mole percent of isoeugenol containing monomeric units in the polymer.

**Figure 2:**
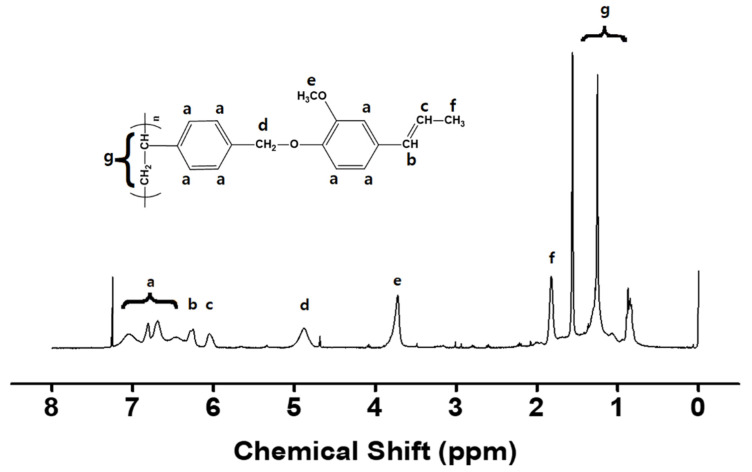
^1^H NMR (proton nuclear magnetic resonance) spectrum of PIEU100.

Figure 1 shows the synthetic routes to the isoeugenol-substituted polystyrenes (PIEU100) and copolymers (PIEU80, PIEU60, PIEU40, and PIEU20, where # is the molar content (%) of isoeugenol side groups). Copolymers with different degrees of substitution were obtained by varying the amount of isoeugenol in the reaction as shown in Table 1. Almost 100% conversion of chloromethyl to isoeugenyl methyl ether was obtained when 150 mol% of isoeugenol was used at 70 °C for 24 h, as shown by assignment of the respective proton peaks of the isoeugenol-containing homopolymer (PIEU100) (Figure 2). Figure 2 shows the proton nuclear magnetic resonance (^1^H NMR) spectrum and assignment of the respective peaks of PIEU100, confirming the chemical composition of the monomeric units in the obtained polymers. The spectrum indicates the presence of protons from the phenyl ring of the styrene backbone (*δ* = 6.3–7.2 ppm (peak a)). The proton peaks from the isoeugenol side chains (*δ* = 6.1–6.3 (peak b), 5.9–6.1 (peak c), 3.5–3.9 (peak e), and 1.6–1.8 (peak f) indicate the inclusion of isoeugenol moieties in the polymer. The content of isoeugenol can be calculated by comparing the integrated area of the proton peaks of the isoeugenol side chain (*δ* = 3.5–3.9 ppm, peak e) and chloromethyl side chains (*δ* = 4.6–5.1 ppm, peak d). Similar integrations and calculations for PIEU80, PIEU60, PIEU40, and PIEU20 were performed and were typically within ±10% of the expected values from the synthesis. These polymers have good solubility in medium-polarity solvents with low boiling temperatures, such as chloroform and tetrahydrofuran, as well as in aprotic polar solvents, such as *N*,*N*′-dimethylacetamide (DMAc), *N*,*N*′-dimethylformamide (DMF), and *N*-methyl-2-pyrrolidone (NMP). The good solubility of all polymers in common solvents is of use in fabrication for film applications.

**Figure 3:**
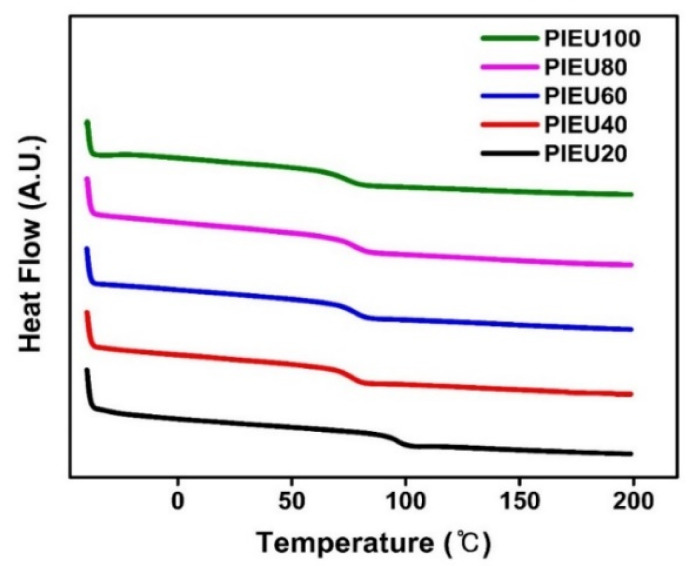
DSC (differential scanning calorimetry) thermogram of isoeugenol-modified polystyrene (PIEU#).

The thermal characteristics of the synthesized polymers were investigated using differential scanning calorimetry. All the samples were amorphous materials, as only one glassy-to-rubbery transition behavior was observed in the differential scanning calorimetry thermogram (Figure 3). As the molar content of the isoeugenol side group increased from 20% to 100%, the *T*_g_ value decreased from 96 °C for PIEU20 to 74 °C for PIEU100. The decrease in the *T*_g_ value of the polystyrene derivatives with increasing molar content of the bulky side group has been previously reported and was ascribed to an increase in the free volume that is the space present inside the polymer, as polymers with larger free volumes have lower *T*_g_ values [81,82]. Additionally, the *T*_g_ value of the PS derivatives depends on the interplay of the free volume inside the polymer and physico-chemical interactions among the polymer chains [83]. Therefore, the trend of the *T*_g_ value is interpreted with the other two analysis points: free volume effect and interaction effect. For example, in this study, from PIEU20 to PIEU40, the *T*_g_ value decreased dramatically because the free volume effect was dominant. When the molar content of the isoeugenol containing monomer units in PIEU# was greater than about 40 mol% (PIEU40, PIEU60, PIEU80, and PIEU100), the difference in *T*_g_ values was subtle because the interaction among the isoeugenol side chains was also expressed.

### 3.2. LC Orientation Behavior of the LC Cell Fabricated with Isoeugenol-Modified Polystyrene Film

**Figure 4:**
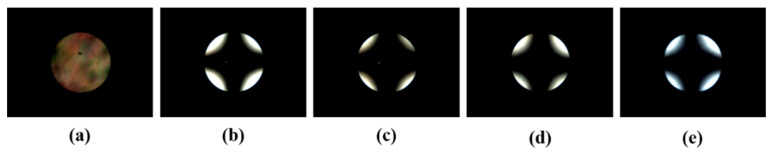
Conoscopic polarized optical microscopy images of the liquid crystal (LC) cells fabricated with PIEU100 films under the following weight ratios of PIEU100: (**a**) 0.001, (**b**) 0.01, (**c**) 0.05, (**d**) 0.1, and (**e**) 1.0 wt.%.

Figure 4 shows conoscopic polarized optical microscopy (POM) images of the LC cells fabricated with PIEU100 films on glass substrates at the following weight concentrations of PIEU100: 0.001, 0.01, 0.05, 0.1, and 1.0 wt.%. At first, as shown in Figure 4a, random planar alignment was observed at a PIEU100 weight ratio of 0.001 wt.%. When PIEU100 weight ratios in solution were more than 0.01 wt.%, vertical LC aligning behavior was observed, as shown in the Maltese cross-pattern in conoscopic POM images (Figure 4b–e). Therefore, a 1 wt.% solution was selected as a coating solution to fabricate LC cells made from PIEU# films, as previously reported by other researchers [84].

**Figure 5:**
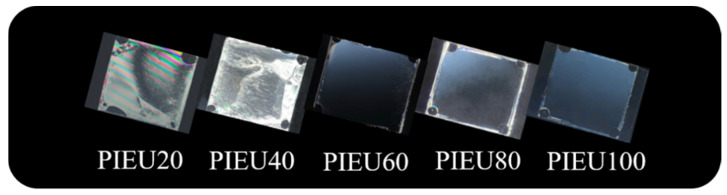
Photograph images of the LC cells made from PIEU# films according to the molar content of isoeugenol moiety.

Figure 5 shows photographic images of LC cells made from the PIEU# copolymers. The LC cells fabricated with PIEU# films with an isoeugenol side group content of less than 40 mol% (PIEU40) partially showed LC texture with birefringence, while good uniformity of vertical LC alignment behavior was observed for LC cells fabricated with the polymer films with an isoeugenol side group content of at least 60 mol% (PIEU60, PIEU80, and PIEU100) in the entire area image area. All of the PIEU100 films could induce stable vertical LC aligning behaviors, and the vertical LC alignment was sustained for at least several months. Therefore, as the content of the isoeugenol side groups increased, the vertical aligning capability of the LC cells made from polymers increased.

**Figure 6:**
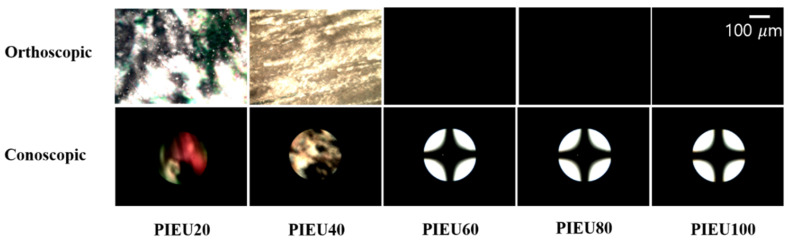
Orthoscopic and conoscopic polarized optical microscopy images of the LC cells made from PIEU# (PIEU20, PIEU40, PIEU60, PIEU80, and PIEU100) films.

The LC aligning behaviors of the LC cells made from PIEU# films were also examined by observing orthoscopic and conoscopic POM images, as shown in Figure 6. Random planar LC aligning behavior was observed for LC cells made with the poly(4-chloroemthylstyrene) film (figure not shown). When the content of the isoeugenol-containing monomeric part in PIEU# was 20 and 40%, the LC cells fabricated using the PIEU# film exhibited random planar LC alignment behavior in the orthoscopic and conoscopic POM images. On the other hand, vertical LC aligning behavior was observed for the LC cells made with the polymeric films PIEU60, PIEU80, and PIEU100, as can be seen in the Maltese cross-pattern of the orthoscopic and conoscopic POM images.

### 3.3. Surface Properties of Isoeugenol-Modified Polystyrene Films

**Figure 7:**
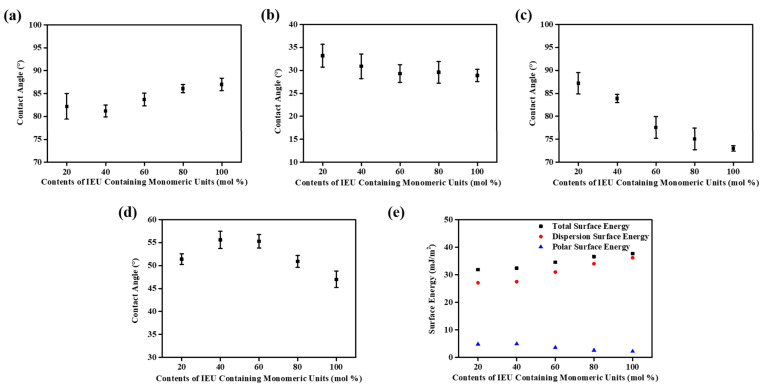
Contact angles of (**a**) water, (**b**) diiodomethane, (**c**) formamide, (**d**) ethylene glycol, and (**e**) surface energy values of PIEU# films according to the molar content of the isoeugenol moiety in the side groups.

Based on the results of inducing LC alignment properties, we observed a general trend that polymers containing higher contents of the isoeugenol side group possessed a preference for vertical alignment of LC molecules. It has been known that the high tilt angles of LCs that produce vertical aligning properties are well correlated with low surface energy values on the alignment film and/or steric repulsion between LCs and the alignment surfaces [85,86]. For instance, polyimide derivatives having bulky and nonpolar groups such as 4-*n*-octyloxyphenyloxy and pentylcyclohexylbenzene exhibited vertical aligning behavior [53]. Thus, we tried to explain the aligning behavior of the LC molecules on the PIEU# films using several surface characterization techniques, viz. surface energy value measurements. Figure 7 and Table 2 show the surface energy values of the polymer films obtained on the basis of static contact angles of water, methylene iodide, formamide, and ethylene glycol. The total surface energy was calculated using the Owens–Wendt equation, which is a summation of the polar and dispersion contributions. We also found that there is a critical surface energy value of the polymers that provides vertical LC alignment behavior. Vertical LC alignment was observed in the PIEU60, PIEU80, and PIEU100 films. The polar surface energy values of these polymers were ≤3.59 mJ/m^2^: (PIEU60—3.59 mJ/m^2^, PIEU80—2.62 mJ/m^2^, and PIEU100—2.18 mJ/m^2^), whereas the PIEU20 and PIEU40, which have polar surface energy values ≥4.81 mJ/m^2^, did not show vertical alignment behavior. Therefore, it is appropriate to conclude that the vertical aligning capability of PIEU60, PIEU80, and PIEU100 was due to enhanced steric repulsion between LCs and polymeric surfaces caused by introducing bulky and nonpolar isoeugenol moieties into the side chain of polystyrene, and due to the low polar surface energy (≤3.59 mJ/m^2^) originating from the peculiar molecular structure of the nonpolar carbon containing groups.

### 3.4. Reliability and Electro-Optical Performance of the LC Cells Fabricated with Isoeugenol-Modified Polystyrene Films

**Figure 8:**
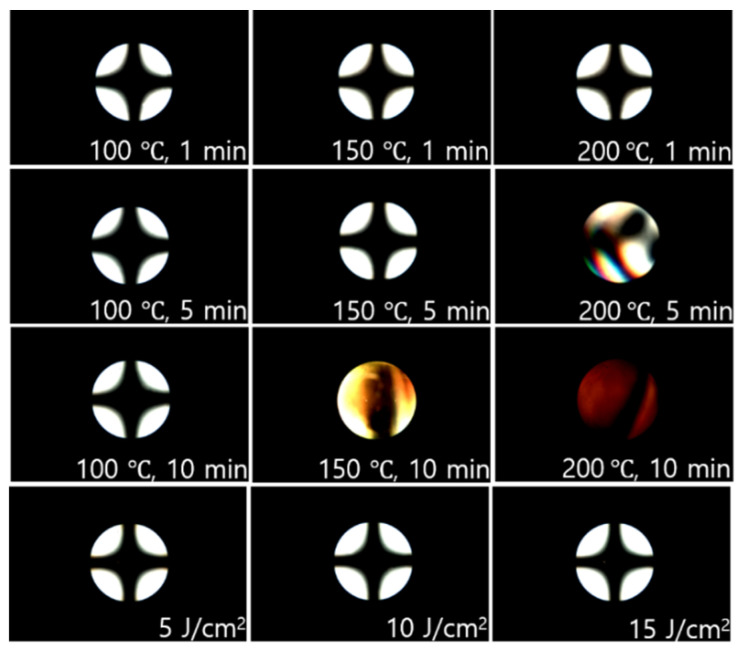
Concoscopic polarized optical microscopy images of the LC cells made using PIEU100 films, after thermal treatment at 100, 150, and 200 °C for 1, 5, and 10 min, and UV treatment at 5, 10, and 15 J/cm^2^, respectively.

The reliability of the LC cells made from the polymer films was investigated through a stability test of the LC alignment under harsh conditions such as high temperatures and ultraviolet (UV) energy. The thermal and UV stabilities of the LC cell made from the PIEU100 film were estimated from the POM image after heating for 1, 5, and 10 min at 100, 150, and 200 °C, and UV irradiation at 5, 10, and 15 J/cm^2^, respectively. As shown in Figure 8, no significant difference in the pretilt angle on the PIEU100 film with vertical LC alignment ability was observed through the Maltese cross-pattern in the conoscopic POM images, indicating that the vertical LC alignment ability of the PIEU100 LC cell was maintained even at a high temperature of 100 °C and an UV energy of 15 J/cm^2^. The total surface energy values of the PIEU100 films obtained on the basis of the static contact angles of water, methylene iodide, formamide, and ethylene glycol were also measured after heating and UV irradiation. When the temperature was increased to 100 °C and the UV energy was increased to 15 J/cm^2^, the polar surface energy value of the PIEU100 film was maintained in the range 2.0–2.4 mJ/m^2^. Therefore, PIEU# with renewable resources can be considered as a candidate LC alignment layer for eco-friendly applications.

## 4. Conclusions

A series of polystyrene derivatives containing plant-based isoeugenol (PIEU#) was synthesized to investigate the liquid crystal (LC) alignment properties of these polymer films. LC cells made from films of the polymers with ≥60 mol% of isoeugenol units (PIEU60, PIEU80, and PIEU100) showed vertical LC alignment. However, LC cells made from PIEU# films with 40 mol% or less of isoeugenol exhibited random planar LC alignment behavior. The vertical LC alignment was ascribed to steric repulsion between the LC molecules and the polymer surface owing to the incorporation of a nonpolar and bulky isoeugenol moiety into the side chain, and it was well correlated with polar surface energy values of the polymer ≤3.59 mJ/m^2^, generated by the long alkyl groups. This provides a concept for the design of eco-friendly LC alignment layers based on renewable bioresource-containing polymer films.

## Figures and Tables

**Table 1:** Reaction conditions and results for the synthesis of isoeugenol modified polystyrene (PIEU#).

Polymer Designation	Feed Ratio of Isoeugenol [mol%]	Degree of Substitution [%]	*T*_g_ [°C]
PIEU20	20	20	96
PIEU40	40	40	76
PIEU60	60	60	77
PIEU80	80	80	77
PIEU100	150	100	74

**Table 2:** Surface energy values and liquid crystal (LC) alignment properties.

Polymer Designation	Contact Angle (°) ^a^				Surface Energy (mJ/m^2^) ^b^			LC Aligning Ability ^c^
	Water	Diiodo Methane	Formamide	Ethylene Glycol	Polar	Dispersion	Total	
PIEU20	82.2	33.2	87.2	51.4	4.8	27.1	31.9	X
PIEU40	81.3	30.9	83.9	55.6	4.9	27.5	32.4	X
PIEU60	83.7	29.3	77.6	55.3	3.6	31.0	34.6	O
PIEU80	86.1	29.6	75.1	50.9	2.6	34.0	36.6	O
PIEU100	87.1	28.9	73.0	47.0	2.2	36.2	38.4	O

^a^ Measured from static contact angles. ^b^ Calculated from Owens–Wendt’s equation. ^c^ Circle (O) and cross (X) indicate polymer films have vertical and random planar, tilted LC aligning ability, respectively.

## Data Availability

The data presented in this study are available on request from the corresponding author.
